# Long-Term Effect of Acupuncture on the Medical Expenditure and Risk of Depression and Anxiety in Migraine Patients: A Retrospective Cohort Study

**DOI:** 10.3389/fneur.2020.00321

**Published:** 2020-04-24

**Authors:** Chung-Chih Liao, Ke-Ru Liao, Cheng-Li Lin, Jung-Miao Li

**Affiliations:** ^1^Graduate Institute of Chinese Medicine, College of Chinese Medicine, China Medical University, Taichung, Taiwan; ^2^Department of Neurology, Yuanlin Christian Hospital, Yuanlin, Taiwan; ^3^Management Office for Health Data, China Medical University Hospital, Taichung, Taiwan; ^4^College of Medicine, China Medical University, Taichung, Taiwan; ^5^Department of Chinese Medicine, Show Chwan Memorial Hospital, Changhua, Taiwan

**Keywords:** acupuncture, migraine, medical expenditure, depression, anxiety

## Abstract

**Background:** Migraine, a common neurological disorder, increases the risk of psychiatric disorders. Currently, the efficacy of conventional therapies is considered unsatisfactory. Acupuncture has been gaining popularity in treatment of neuropsychiatric disease. This study aimed to investigate the effect of acupuncture on medical expenditure and the risk of depression and anxiety in migraine patients.

**Methods:** Patients with migraine were either selected for acupuncture treatment or no treatment based on the 1:1 propensity score-matched method from the Taiwanese National Health Insurance Research Database between 2000 and 2012 and followed up until the end of 2013. The observed outcome measures were comparison of medical expenditure and incidence of depression/anxiety in the two cohorts.

**Results:** The acupuncture cohort had a lower medical expenditure within 1 year of the intervention than the non-acupuncture cohort (*p* < 0.001). The acupuncture cohort had a reduced risk of depression [adjusted hazard ratio [HR], 0.61, 95% confidence interval [CI], 0.39–0.95] and anxiety (adjusted HR, 0.51, 95% CI, 0.43–0.59) after adjusting for sex, age, monthly income, urbanization level, occupation category, baseline comorbidities, and medicines used. The Kaplan–Meier analysis revealed that the cumulative incidence of depression and anxiety was significantly lower in the acupuncture cohort than in the non-acupuncture cohort during the 13-year follow-up period (log-rank test, *p* < 0.001).

**Conclusions:** Acupuncture could reduce medical expenditure and the risk of depression and anxiety during the long-term follow-up period in migraine patients. However, the regulatory effects and mechanisms should be assessed in further clinical research.

## Introduction

Migraine is a common neurological disease. It is the second most disabling disease in the world, and affects about 1/10 of the human population, with an especially dominant prevalence in females, students, and urban residents (1, 2). Migraine often interferes with patient's daily activities and causes a considerable burden to the medical cost. According to a report, migraine causes an estimated annual loss of 17 billion dollars in the United States (3). Recently, psychiatric disorders have increased in prevalence worldwide, leading to higher mortality and reduced quality of life for those affected. These disorders are frequently comorbid with migraine, especially migraine chronification. Among them, depression and anxiety are the most frequent psychiatric disorders associated with migraine (4, 5). Migraine has a definitive bi-directional relationship with depression and anxiety (6, 7). Migraine patients comorbid with depression and anxiety would impact on patient's quality of life, work performance, and family burden. Suitable treatment is urgently required to solve this clinical problem (8).

Although conventional Western medicine has developed convenient treatments for migraines, the average efficacy rate of prophylactic drugs is still poor and may result in intolerable adverse effects (9). Medications also exhibit limited efficacy to prevent psychiatric disorders. Hence, numerous non-pharmacological treatments, such as neuro-stimulation or other newly developed complementary therapies, are becoming increasingly popular and accepted (9, 10).

Acupuncture has been applied to neuropsychiatric disorders throughout Asia for more than 2000 years. It helps patients to improve health by mainly regulating Yin and Yang balance through meridians and causes beneficial analgesic effects. Recently, acupuncture was shown to be an effective therapy for reducing pain severity in acute migraine attack or prevention of frequent and chronic migraine (11–16). However, there is still scarce evidence in long-term follow-up regarding whether acupuncture could reduce medical expenditure or decrease the risk of depression and anxiety development in the migraine population. Therefore, more relevant real-world data is needed to discern this treatment's efficacy.

All public insurance systems were integrated into the National Health Insurance (NHI) program in Taiwan in 1995 as a single-pay program to serve all residents, with 99% of 23.74 million residents covered in 2009. It has offered insurance cover for Traditional Chinese Medicine (TCM, including; Chinese herbal medicines, acupuncture, and traumatology manipulative therapies) and conventional Western medicine (17, 18). Intact registration files and original claim data of acupuncture utilization were collected in the Taiwanese National Health Insurance Research Database (NHIRD). This database is considered to reflect a realistic acupuncture clinical practice for research.

Therefore, the present study aimed to investigate the cost benefit of acupuncture in migraine and subsequent psychiatric disorders. The effectiveness of acupuncture on reducing migraine medical cost and risk of depression and anxiety development in Taiwan was analyzed using the NHIRD information.

## Materials and Methods

### Data Source

We conducted a nationwide, population-based, propensity score-matched cohort analysis by using the Longitudinal Health Insurance Database 2000 (LHID 2000), which comprises a random sample of one million subjects from the NHIRD longitudinally linked data available from 1997 through 2013. These files were linked by using a scrambled, anonymous identification number for each subject to obtained longitudinal medical history. The *International Classification of Diseases, Ninth Revision, Clinical Modification* (ICD-9-CM) was used for the diagnosis codes. This study was approved by the Institutional Review Board of China Medical University in central Taiwan (CMUH-104-REC2-115-R4).

### Study Subjects

**Figure 1:**
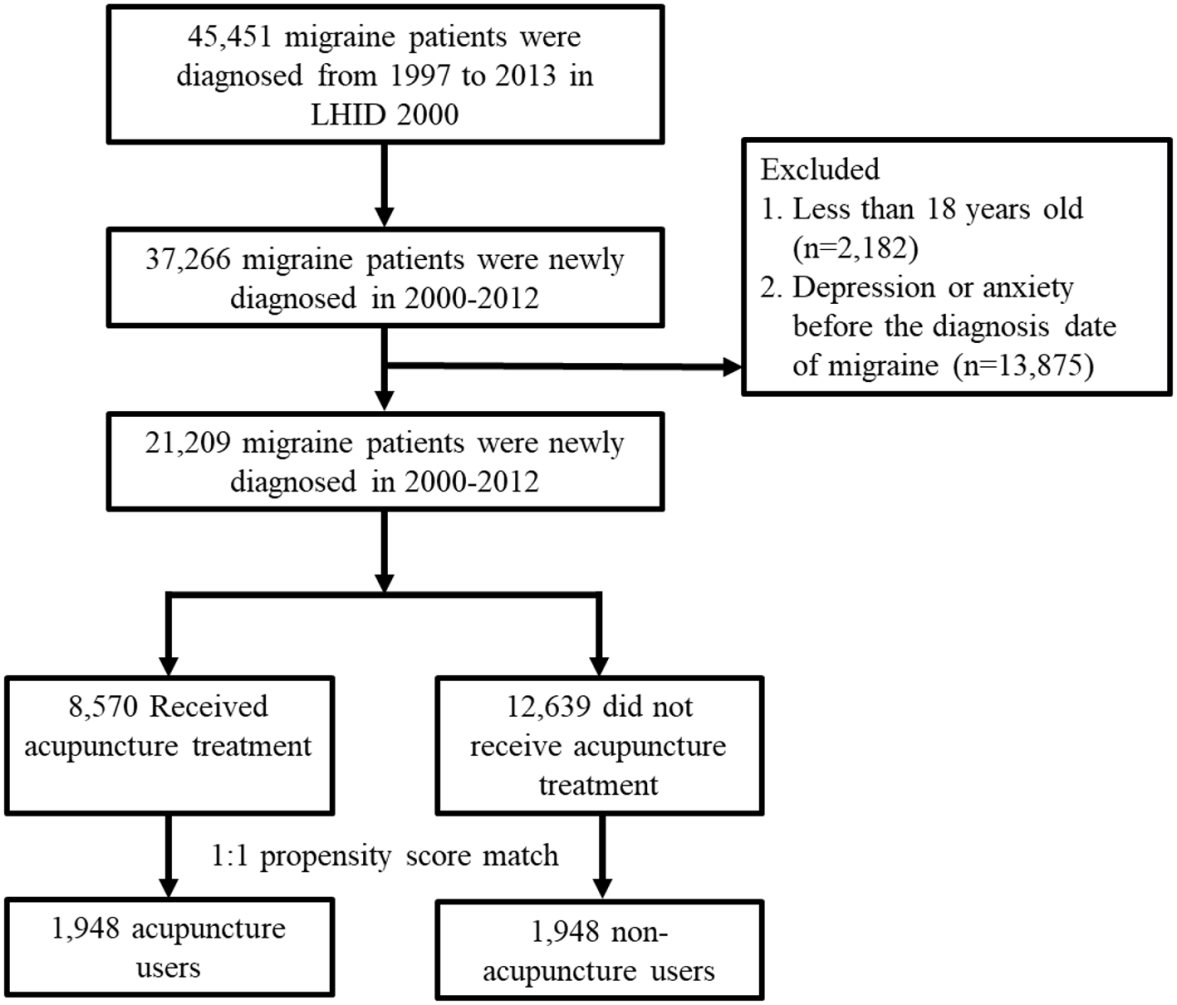
Flowchart for study subject enrolment. LHID 2000: Longitudinal Health Insurance Database 2000.

We included all patients diagnosed with migraine (ICD-9-CM: 346), which at least two ambulatory or inpatient claims from January 1, 2000 to December 31, 2012 in the LHID 2000. The exclusion criteria were age <18 years old, and depression (ICD-9-CM: 296.2, 296.3, 300.4, 311) or anxiety (ICD-9-CM: 300) before the diagnosis date of migraine. Patients who had received acupuncture treatment after the new diagnosis date of migraine were defined as the acupuncture cohort. The date of first acupuncture after new diagnosis date of migraine was defined as index date for acupuncture cohort. The index date for non-acupuncture patients were randomly appointed a month and day with the same index year of the matched acupuncture cases. The 1:1 propensity score method by sex, age, income, urbanization, occupation, baseline comorbidities, conventional medicine used, and diagnosis year of migraine and index year was used to match an equal number of patients in both cohorts. The flowchart was summarized in Figure 1. The follow-up period was from the index date to the patient removed from the NHI program, the patient experienced a depression or anxiety event, or December 31, 2013.

### Covariate Assessment

Covariates considered in our study include sociodemographic factors, baseline comorbidities, and medicines. Sociodemographic factors included sex, age, monthly income, urbanization level, and occupation categories. Baseline comorbidities at least two ambulatory or inpatient claims were considered exist, including diabetes mellitus (ICD-9-CM 250), hypertension (ICD-9-CM 401–405), hyperlipidaemia (ICD-9-CM 272), stroke (ICD-9-CM 430–438), coronary artery disease (ICD-9-CM codes 410–414), mental disorders (ICD-9-CM 290–319 except ICD-9-CM: 296.2, 296.3, 300, 311), traumatic brain injury (ICD-9-CM 310.2, 800–804, 850–854, 873.x, 910.x, 920.x, 959.1), Parkinson's disease (ICD-9-CM 332), cirrhosis (ICD-9-CM 571), chronic obstructive pulmonary disease (COPD) (ICD-9-CM 491, 492, 496), renal disease (ICD-9-CM 585). Medicines including conventional medicine (triptans, ergots, single analgestic, and NSAIDs) and Chinese herbal medicine were also considered in this study.

### Outcome Measurement

We analyzed total number of outpatient visits and medical expenditure within 1 year after the index date, as well as incidence rates of depression and anxiety during the study period between the acupuncture cohort and non-acupuncture cohorts with migraine.

### Statistical Analysis

Differences in baseline characteristics between acupuncture users and acupuncture non-users were examined using Chi-squared test for categorical variables and *t*-test for continuous variables. Means of outpatient visits and total medical expenditures between both cohorts were examined using *t*-test. Incidence rates of depression and anxiety were calculated for both cohorts. Cox proportional hazards regression was performed to evaluate the hazard ratios (HRs) with 95% confidence intervals (CIs) after adjusting each covariate. Kaplan–Meier methods and log-rank tests were used to estimate the differences in the cumulative incidence of depression and anxiety during 13-year follow-up period between the two cohorts. *p* < 0.05 indicated statistically significant. All statistical analyses and figures were performed using SAS software, version 9.4 (SAS Institute Inc., Cary, NC, U.S.A.).

## Results

### Demographic Characteristics Between Acupuncture Cohort and Non-acupuncture Cohort

We identified a total of 21,209 patients with newly diagnosed migraine who met the criterion between 1 January 2000 and 31 December 2012 in the LHID 2000 (Figure 1). Among the included subjects, 8,570 patients received acupuncture treatment, and 12,639 patients did not receive acupuncture treatment from the initial migraine diagnosis date to 31 December 2012. We ultimately used a 1:1 propensity-score matching method to randomly select 1,948 patients in the acupuncture cohort and non-acupuncture cohort, in order to minimize the interference of confounding variables.

The baseline characteristics of both cohorts are presented in Table 1. The proportion of women was about 68%, and the mean age was about 44 years. After the matching procedure, there were no significant differences in sex, age, urbanization, occupation, baseline comorbidities, and conventional medicine used between migraine patients with or without receiving acupuncture treatment. In addition, the proportion of Chinese Herbal medicine used in the acupuncture group was significant higher than the non-acupuncture group (98.4 vs. 87.0%, *p* = 0.001).

**Table 1:** Characteristics of migraine patients according to reception of acupuncture.

**Variable**	**Received acupuncture**				*p*-value^*^
	**No (*****n*** **=1948)**		**Yes (*****n*** **=1948)**		
	***n***	**%**	***n***	**%**	
Sex					0.49
Female	1342	68.9	1322	67.9	
Male	606	31.1	626	32.1	
Age group					0.10
18–29	509	26.1	503	25.8	
30–39	381	19.6	367	18.8	
40–49	406	20.8	400	20.5	
50–59	244	12.5	293	15.0	
60–69	208	10.7	223	11.5	
≥70	200	10.3	162	8.32	
Mean ± SD (years)^†^	44.2 ± 17.3		44.1 ± 16.6		0.85
Monthly income^***^					0.03
<15,000 NTD (<500 USD)	879	45.1	796	40.9	
15,000–19,999 NTD (500-666.6 USD)	500	25.7	538	27.6	
≥ 20,000 NTD (≥ 666.66 USD)	569	29.2	614	31.5	
Urbanization level^&^					0.05
1 (highest)	544	27.9	582	29.9	
2	571	29.3	593	30.4	
3	317	16.3	331	17.0	
4 (lowest)	516	26.5	442	22.7	
Occupation category^‡^					0.21
Office worker	1017	52.2	1072	55.0	
Laborer	761	39.1	719	36.9	
Other	170	8.73	157	8.06	
Baseline Comorbidity					
Diabetes mellitus	103	5.29	113	5.80	0.48
Hypertension	629	32.3	647	33.2	0.54
Hyperlipidaemia	414	21.3	418	21.5	0.88
Stroke	63	3.23	59	3.03	0.71
Coronary artery disease	335	17.2	314	16.1	0.37
Mental disorders	216	11.1	228	11.7	0.55
Traumatic brain injury	301	15.5	324	16.6	0.32
Parkinson's disease	22	1.13	16	0.82	0.33
Cirrhosis	463	23.8	494	25.4	0.25
COPD	282	14.5	268	13.8	0.52
Renal disease	38	1.95	25	1.28	0.10
Conventional medicine					
Triptans	28	1.44	34	1.75	0.44
Ergots	622	31.9	576	29.6	0.11
Single analgestic	1938	99.5	1935	99.3	0.53
NSAIDs	1922	98.7	1920	98.6	0.78
Chinese herbal medicine	1305	87.0	1917	98.4	0.001
Duration between migraine date and index, days (mean, median)	(905.8, 574)		(922.8, 571)		

*The mean (median) of the follow-up period for anxiety was 4.78 (3.39) and 2.60 (2.74) years for acupuncture cohort and compared cohort, respectively; the mean (median) of the follow-up period for depression was 5.50 (3.60) and 4.03 (3.64) years for acupuncture cohort and compared cohort, respectively*.

* Chi-square test;

† *t-test*.

*** *New Taiwan Dollar (NTD), 1 NTD is equal to 0.03 USD*.

& *The urbanization level was divided by the population density of the residential area into 4 levels, which level 1 was the most urbanized and level 4 was the least urbanized*.

‡ *Other occupation categories included those who were primarily retired, unemployed, and low-income populations*.

The mean duration between the initial diagnosis of migraine and the first receiving of acupuncture was approximately 923 days. The mean follow-up periods for depression were 5.50 and 4.03 years for the acupuncture cohort and non-acupuncture cohort, respectively. The mean follow-up periods for anxiety were 4.78 and 2.60 years for the acupuncture cohort and non-acupuncture cohort, respectively.

### The Effect of Acupuncture on the Total Medical Expenditure During a One-Year Follow-Up Period With Migraine

The number of outpatient visits and total medical expenditure of the acupuncture users and non-users with migraine for the 1-year follow-up period after the index/pseudo index date is compared in Table 2. Acupuncture users had significantly more outpatient visits than non-users (*p* < 0.001). However, acupuncture users had significantly lower total medical expenditures (*p* < 0.001) compared with non-users.

**Table 2:** Comparisons of number of outpatient visits and total medical expenditures between acupuncture users and acupuncture non-users within 1 year after index date (the date of first receiving acupuncture treatment).

**Variable**	**Received Acupuncture**		***p*-value^†^**
	**No (*n* = 1948)**	**Yes (*n* = 1948)**	
**Outpatient visits (mean** **±** **SD)**	21.7 ± 19.2	29.6 ± 22.0	<0.001
Total medical expenditures, NTD (mean ± SD)	20863.4 ± 102835.3	17039.6 ± 80545.8	<0.001

*SD, standard deviation*.

† *t-test*.

### The Effect of Acupuncture on the Depression and Anxiety Development During a Thirteen-Year Follow-Up Period With Migraine

Among the patients with migraine during the follow-up period, 40 patients in the acupuncture cohort (3.74 per 1000 person-years) and 54 patients in the non-acupuncture cohort (6.88 per 1000 person-years) developed depression. Three hundred and thirty-six patients in the acupuncture cohort (36.1 per 1000 person-years) and 548 patients in the non-acupuncture cohort (108.2 per 1000 person-years) developed anxiety (Table 3). After adjustment for sex, age, monthly income, urbanization level, occupation category, baseline comorbidities and medicines, migraine patients receiving acupuncture treatment were less likely to develop both depression and anxiety compared to those not receiving acupuncture treatment (adjusted HR, 0.61, 95% CI, 0.39–0.95; adjusted HR, 0.51, 95% CI, 0.43–0.59, respectively) (Tables 3, 4).

**Table 3:** Incidence rates, hazard ratio, and confidence intervals of depression and anxiety for migraine patients who did and did not receive acupuncture treatment.

**Variables**	**Received acupuncture**						**Compared with non-acupuncture users**	
	**No (*****n*** **=** **1948)**			**Yes (*****n*** **=** **1948)**			**Crude HR**	**Adjusted HR^**†**^**
	**Event**	**Person years**	**IR**	**Event**	**Person years**	**IR**	**(95%CI)**	**(95%CI)**
**Depression**	54	7,850	6.88	40	10,709	3.74	0.61(0.41, 0.92)^*^	0.61(0.39, 0.95)^*^
**Anxiety**	548	5,065	108.2	336	9,304	36.1	0.40(0.35, 0.46)^***^	0.51(0.43, 0.59)^***^

*IR, incidence rates, per 1,000 person-years; HR, hazard ratio; CI, confidence interval*.

† *Adjusted HR: adjusted for receiving acupuncture, age, sex, monthly income, urbanization level, occupation category, diabetes mellitus, hypertension, hyperlipidaemia, stroke, coronary artery disease, and mental disorders, traumatic brain injury, Parkinson's disease, cirrhosis, COPD, renal disease, medicine of triptans, ergots, single analgestic, NSAIDs, and Chinese herbal medicine in Cox proportional hazards regression*.

* p < 0.05;

*** *p < 0.001*.

**Table 4:** Hazard ratios of depression and anxiety with covariates among patients with migraine in multivariable Cox proportional hazards regression.

	**Depression**		**Anxiety**	
**Variable**	**Crude HR^**†**^ (95% CI)**	**Adjusted HR^**‡**^ (95% CI)**	**Crude HR^**†**^ (95% CI)**	**Adjusted HR^**‡**^ (95% CI)**
**Received acupuncture**	0.61(0.41, 0.92)^*^	0.61(0.39, 0.95)^*^	0.40(0.35, 0.46)^***^	0.51(0.43, 0.59)^***^
**Sex(Male vs. Female)**	1.81(1.09, 2.99)^*^	2.02(1.18, 3.45)^*^	1.29(1.11, 1.50)^***^	1.56(1.33, 1.82)^***^
**Age, years**				
18–29	1.00	1.00	1.00	1.00
30–39	0.97(0.52, 1.81)	0.72(0.35, 1.47)	1.21(0.97, 1.52)	1.05(0.82, 1.35)
40–49	1.17(0.66, 2.07)	0.75(0.36, 1.54)	1.70(1.39, 2.09)^***^	1.42(1.10, 1.82)^***^
50–59	0.68(0.32, 1.47)	0.43(0.17, 1.13)	1.52(1.21, 1.91)^***^	1.19(0.88, 1.60)
60–69	1.08(0.54, 2.16)	0.64(0.26, 1.59)	2.26(1.81, 2.81)^***^	1.82(1.36, 2.42)^***^
≥70	1.11(0.52, 2.38)	0.77(0.28, 2.11)	1.46(1.11, 1.91)^***^	1.27(0.91, 1.77)
**Monthly income**				
<15,000 NTD (<500 USD)	1.00	1.00	1.00	1.00
15,000–19,999 NTD (500-666.6 USD)	1.31(0.79, 2.17)	1.49(0.80, 2.78)	1.53(1.30, 1.80)^***^	1.19(0.96, 1.46)
≥ 20,000 NTD (≥ 666.66 USD)	1.35(0.84, 2.19)	1.67(0.90, 3.11)	1.45(1.23, 1.70)^***^	1.15(0.93, 1.42)
**Urbanization level**				
1 (highest)	1.00	1.00	1.00	1.00
2	1.66(0.96, 2.89)	1.78(1.01, 3.12)^*^	1.04(0.87, 1.24)	1.04(0.87, 1.24)
3	1.46(0.77, 2.79)	1.53(0.79, 2.93)	1.02(0.83, 1.26)	1.05(0.85, 1.29)
4 (lowest)	1.36(0.75, 2.48)	1.40(0.75, 2.62)	1.09(0.91, 1.31)	0.98(0.81, 1.18)
**Occupation category**				
Office worker	1.00	1.00	1.00	1.00
Laborer	0.87(0.57, 1.33)	0.78(0.49, 1.23)	1.19(1.04, 1.36)^*^	1.05(0.90, 1.22)
Other	0.60(0.24, 1.50)	0.69(0.26, 1.84)	0.84(0.64, 1.10)	0.89(0.66, 1.20)
**Baseline comorbidities (yes vs. no)**				
Diabetes mellitus	0.97(0.39, 2.38)	0.88(0.33, 2.33)	0.87(0.64, 1.18)	0.65(0.47, 0.90)^**^
Hypertension	1.15(0.75, 1.74)	1.12(0.63, 1.99)	1.51(1.32, 1.73)^***^	1.17(0.98, 1.39)
Hyperlipidaemia	1.05(0.65, 1.70)	1.15(0.64, 2.05)	1.32(1.14, 1.54)^***^	1.02(0.85, 1.22)
Stroke	1.49(0.55, 4.05)	1.94(0.68, 5.53)	0.67(0.43, 1.06)	0.57(0.36, 0.91)^*^
Coronary artery disease	1.44(0.89, 2.34)	1.64(0.89, 3.05)	1.59(1.35, 1.86)^***^	1.36(1.12, 1.64)^***^
Mental disorders	-	-	-	-
Traumatic brain injury	1.12(0.65, 1.92)	1.27(0.73, 2.19)	1.06(0.89, 1.27)	1.22(1.01, 1.46)^**^
Parkinson's disease	-	-	0.80(0.36, 1.79)	0.91(0.40, 2.05)
Cirrhosis	0.90(0.56, 1.46)	0.90(0.53, 1.50)	1.41(1.22, 1.63)^***^	1.27(1.09, 1.48)^**^
COPD	1.37(0.81, 2.31)	1.55(0.87, 2.76)	1.07(0.89, 1.28)	0.95(0.78, 1.16)
Renal disease	-	-	0.69(0.36, 1.33)	0.52(0.27, 1.02)
**Conventional medicine**				
Triptans	0.84(0.12, 6.05)	1.20(0.17, 8.75)	0.77(0.40, 1.49)	0.97(0.50, 1.89)
Ergots	1.27(0.84, 1.94)	1.27(0.83, 1.96)	1.26(1.09, 1.44)^**^	1.18(1.02, 1.36)^*^
Single analgestic	0.31(0.08, 1.26)	0.31(0.07, 1.43)	1.83(0.69, 4.89)	1.63(0.60, 4.40)
NSAIDs	0.50(0.16, 1.57)	0.47(0.13, 1.62)	1.55(0.85, 2.80)	1.67(0.91, 3.06)
**Chinese herbal medicine**	0.82(0.49, 1.37)	1.04(0.58, 1.84)	0.32(0.28, 0.37)^***^	0.43(0.37, 0.51)^***^

† Crude HR, relative hazard ratio;

‡ *Adjusted HR: multivariable analysis including sex, age, monthly income, urbanization level, occupation category, baseline comorbidities and medicines*.

* p < 0.05;

** p < 0.01;

*** *p < 0.001*.

In the multivariate analysis, males had a higher risk of depression and anxiety incidence than females (adjusted HR, 2.02, 95% CI, 1.18–3.45; adjusted HR, 1.56, 95% CI, 1.33–1.82, respectively). The incidence of anxiety in patients with migraine increased in both age 40-49 and 50-59 groups (adjusted HR, 1.42, 95% CI, 1.10–1.82; adjusted HR, 1.82, 95% CI, 1.36–2.42, respectively). Comorbidity with coronary artery disease (adjusted HR, 1.36, 95% CI, 1.12–1.64), traumatic brain injury (adjusted HR, 1.22, 95% CI, 1.01–1.46), and cirrhosis (adjusted HR, 1.27, 95% CI, 1.09–1.48) also increased risk of anxiety incidence. In addition, participants who took Chinese herbal medicine (adjusted HR, 0.43, 95% CI, 0.37–0.51) were associated with a lower risk of anxiety incidence (Table 4).

**Figure 2:**
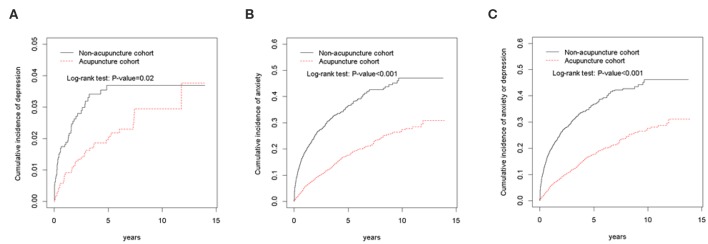
Cumulative incidence of **(A)** depression, **(B)** anxiety, **(C)** combination of depression and anxiety between the acupuncture cohort and the non-acupuncture cohort.

The Kaplan–Meier analysis revealed that the cumulative incidence of anxiety alone, depression alone, and combined anxiety or depression were significantly lower in the acupuncture cohort compared to the non-acupuncture cohort during the 13-year follow-up period (log-rank test, *p* < 0.001, Figure 2).

## Discussion

Through long-term follow-up in a nationwide retrospective cohort study, this is the first report to discover that migraine patients receiving acupuncture treatment could significantly reduce medical expenditure and lower risk of depression/anxiety compared to those not receiving acupuncture treatment.

The NHIRD offered sufficient population sample size and information, eliminating the bias associated with a limited sample size, which was considered closer to real-world evidence and an appropriate source to assess the disease and treatment efficacy. In addition, it is worth noting that the acupuncture technique is only performed by well-trained, qualified TCM doctors in Taiwan, which guarantee the most suitable treatment for patients and accurate, valuable research results.

In the NHIRD randomized sample of patients with migraine, the prevalence was approximately twice as high in women compared with men, and mainly affecting the young and middle-aged population. These results agree with previous studies on this topic (2, 19). According to a large-scale assessment in the United States, patients with migraine imposed a substantial annual economic burden compared with those without migraine (20). Previous studies revealed that the utilization of acupuncture is a common, affordable, and effective treatment in Taiwan (21). In our study, approximately 40.4% of patients with migraine received acupuncture treatment, and acupuncture treatment for patients with migraine could substantially reduce the medical cost within a 1-year period. We assumed the reason might be that acupuncture delivers rapid, safe, effective pain relief, and patients with migraine could reduce medication usage. In addition, acupuncture may reduce the frequency and severity of a migraine attack, which was considered an alternatively effective prophylactic therapy for chronic migraine.

Previous studies reported over a 2.5-fold risk of depression in migraineurs than in the normal population, while anxiety disorders are 2 to 5 times more prevalent in migraineurs than in the healthy population (22). Our data showed that anxiety disorders were much more likely to occur than depression in migraineurs. Following a 13-year survey for migraine cohorts, acupuncture treatment also had a benefit in reducing the incidence of depression/anxiety, as shown by Cox Regression model and Kaplan-Meier method. Migraine and psychiatric disorders share some causative, genetic, biochemical or environmental factors (4, 5, 22). This is the supposed reason as to why acupuncture could simultaneously benefit migraine and depression/anxiety. This finding highlighted how acupuncture might be utilized to eliminate long-term personal, family, negative social activities, and burden beyond migraine itself. In addition, acupuncture as a non-pharmacological therapy may also reduce the chance of medication-overuse headaches, which may arise due to inappropriate use of symptomatic medication for neurological and psychological disorders.

The present study still has some limitations. First, the retrospective nature of our work should be concerned, and a prospective case-control study need to be performed to confirm these data in the future. Second, the NHIRD did not provide the severity, frequency or subtype of migraine. Previous literature has reported that the more frequent the migraine, the stronger the relationship between migraine and depression/anxiety. For example, it has been proved that Chronic Daily Headache (CDH) is associated with higher rates of depressive and anxiety disorders compared to episodic headache (23). Due to the limitation of the database in this retrospective study, we could not answer whether the incidence of depression and anxiety is increased in patients with chronic migraine or in those became chronic during the follow-up period respect to episodic migraineurs. We also could not provide results of acupuncture efficacy for chronic and episodic migraine separately in the present study. Third, the NHIRD data did not contain a symptomatic approach for migraine cohorts. We could not provide how many (percent) of them found acupuncture effective in the acupuncture cohort. In clinical experience, some of patients stopped the acupuncture treatment for migraine for intolerance of pain, trypanophobia or time restriction. In the present study, we also did not have data about acupuncture interruption in our cohort. Furthermore, the information of which acupoints in clinical practice effective to reduce migraine severity or depression/anxiety development could not be acquired in the database. Further rigorous clinical trials or animal experiments are warranted to explain the substantial efficacy and causative mechanism of acupuncture.

## Conclusion

In conclusion, the present study revealed that patients with migraine receiving acupuncture treatment could reduce their medical expenditure and the risk of depression/anxiety development compared to those not receiving acupuncture treatment. The findings support the use of acupuncture as a complementary therapy for migraine which is worthy of being adopted and applied widely.

## Data Availability Statement

The datasets used and analyzed during the current study are available from the corresponding author on reasonable request.

## Ethics Statement

The studies involving human participants were reviewed and approved by Institutional Review Board of China Medical University in central Taiwan (CMUH-104-REC2-115-R4). Written informed consent for participation was not required for this study in accordance with the national legislation and the institutional requirements.

## Author Contributions

C-CL and K-RL interpreted the data and wrote the manuscript. C-LL helped to analyse data from the NHIRD in Taiwan, J-ML designed protocol and revised the manuscript. All authors reviewed the manuscript and agreed to submission.

## Conflict of Interest

The authors declare that the research was conducted in the absence of any commercial or financial relationships that could be construed as a potential conflict of interest.

## Abbreviations

CI: confidential interval
HR: hazard ratio
ICD-9-CM: International Classification of Diseases, Ninth Revision
LHID 2000: Longitudinal Health Insurance Database 2000
NHIRD: National Health Insurance Research Database
TCM: Traditional Chinese medicine.
